# A telemetry dataset on resource utilisation and power consumption in the edge-cloud continuum

**DOI:** 10.1016/j.dib.2026.112734

**Published:** 2026-03-27

**Authors:** Ioanna Angeliki Kapetanidou, Thanasis Kotsiopoulos, Giorgos Thanasoulis, Paschalis Bizopoulos, Athanasios Liatifis, Manolis Skoularikis, Alexandros Nizamis, Panagiotis Sarigiannidis, Konstantinos Votis

**Affiliations:** a Information Technologies Institute, Centre for Research & Technology Hellas, P.O.Box 60361, 6th km Harilaou, Thermi, GR 57001, Thessaloniki, Greece; b Laboratory of Internet of Things and Applications (ITHACA), Department of Electrical and Computer Engineering, University of Western Macedonia, Campus, ZEP Area, Kozani 50100, Greece

**Keywords:** Computing continuum, Kubernetes, Infrastructure monitoring, Energy, Performance, Workloads, Edge device

## Abstract

This paper presents a telemetry dataset capturing resource utilization and power consumption metrics across the ENACT edge-cloud continuum.

The dataset contains empirical telemetry collected in real-time for both infrastructure nodes and application workloads. More specifically, a distributed weather forecasting scenario has been emulated, comprising five pods: two different weather data sources, two forecasting services (one per node/computing layer) and one long-term storage pool. A cloud-based machine and an edge device belonging to the same Kubernetes cluster have been considered for the deployment of the application pods, corresponding to heterogeneous computing tiers.

Data acquisition was performed using ENACT’s Telemetry Data Collector and Monitoring Engine which measures telemetry and energy metrics at node and pod levels in real-time. The resulting dataset provides time-series records including CPU, memory and disk utilization, network throughput, and energy consumption for the cloud node, the edge node and the five application pods.

Telemetry data was collected during two distinct phases: for a period with application workloads running normally and for a baseline period when applications were removed from the cluster. This allows for assessing the impact of the applications activity in terms of resource usage and energy consumption.

This dataset offers valuable insights for the research community in distributed systems, the edge-cloud continuum and cognitive computing, wherein datasets on real-world data, especially reflecting both infrastructure-level and application-level telemetry, are currently very limited. It is particularly useful for developers and research scientists that require such data for tasks such as training and fine-tuning time-series forecasting models, benchmarking anomaly detection models and validating scheduling algorithms and energy-aware strategies, to name a few.

Specifications Table

**Table:** 

Subject	Computer Sciences
Specific subject area	Cloud computing; Edge computing; Cognitive computing; Distributed systems; Telemetry; Continuum monitoring;
Type of data	Table (.csv format) Processed (Aggregated data)
Data collection	Data were collected from a Kubernetes (K8s) cluster deployed on private premises. Telemetry data acquired correspond to two nodes (one located at the cloud and one at the edge) and 5 different K8s pods. Data were obtained during two distinct periods: from the 24th of January 2026 to the 26th of January 2026, when the application pods were running normally, and from the 31st of January 2026 to the 2nd February 2026) when the pods were scaled down to zero replicas to capture baseline system behaviour. Data collection was performed using ENACT’s Telemetry Data Collection and Monitoring Engine and recorded telemetry measurements were stored in the project’s Data Space.
Data source location	The Centre for Research & Technology, Hellas, Thessaloniki, Greece
Data accessibility	Repository name: Zenodo Data identification number: https://doi.org/10.5281/zenodo.18920397 Direct URL to data: https://zenodo.org/records/18920397
Related research article	None

## 1 Value of the Data

• The present dataset contributes to the field of edge-cloud computing wherein telemetry data, although essential for the community, are currently lacking or are not openly accessible. To the best of our knowledge, this is the first dataset providing data for both key resource utilization metrics (CPU/memory/disk usage, network I/O) and energy consumption, obtained at real-time simultaneously at infrastructure-level (involving both cloud and edge nodes, i.e., nodes with diverse computing capabilities) and deployment-level (for pods deployed across the heterogeneous nodes).
• The provided telemetry of resource and energy metrics have been measured in a lifelike cloud-edge setting, providing empirical insights, valuable for cloud, edge, IoT and AI engineers, researchers and developers.
• The dataset will be used for further evaluation of DRL + GNN module utilized to optimize resource allocation in the computer continuum. The initial version of the module is available and published in [1].
• The collected data can be used as a benchmark to train AI models for performance predictions, anomaly detection, etc.
• The dataset can be used for evaluating methods designed for tasks such as resource optimization, energy-efficient orchestration and adaptive scaling across the computing continuum.

## 2 Background

This dataset has been generated in the context of the ENACT project [2,3] to address the need for real-world telemetry capturing resource and power metrics at both workload (pod) and infrastructure (node) levels across heterogeneous environments, including high-capacity cloud nodes and resource-constrained edge devices. Within the project, such data were required for the fine-tuning and training of time series forecasting models and for supporting ENACT’s cognitive scheduling mechanisms, which optimise workload placement based on factors like expected CPU and memory usage and energy impact.

However, to the best of our knowledge, publicly available datasets providing real-world resource utilization and power consumption telemetry, simultaneously at workload and infrastructure levels across heterogeneous edge-cloud settings are currently lacking. Most existing open datasets are either limited to cloud environment [4], focus solely on synthetic workloads [5], or provide aggregated rather than time-series granular measurements [6], which significantly restricts their usefulness.

By addressing this gap, the present dataset is intended to serve as a valuable asset for researchers in distributed systems and AI who require empirical telemetry to evaluate and validate their tools and algorithms, especially for tasks such as time-series forecasting, workload orchestration across the edge-cloud continuum, anomaly detection, and energy-aware computing.

## 3 Data Description

The dataset includes telemetry data from two types of computing nodes:

• vm-node: a cloud-based virtual machine
• rpi4: edge device (Raspberry Pi 4)

Five application workloads (Kubernetes pods) are monitored:

1. Weather data collector (weather data source from Thessaloniki)
2. Weather data collector (weather data source from Berlin)
3. AI-based weather forecaster (edge deployment)
4. AI-based weather forecaster (cloud deployment)
5. A storage pod

Telemetry data are provided for two operational periods:

• 24, 25, and 26 of January 2026, during which all pods were active
• 31 January to 2 February 2026, during which pods were scaled down to zero replicas

The dataset consists of four CSV files:

1. *node_telemetry_pods_on.csv* contains time-series measurements collected from infrastructure nodes for the first period (24/01/2026–26/01/2026), when pods were up
2. *pod_telemetry_pods_on.csv* contains time-series measurements collected by monitoring the deployed pods for the first period (24/01/2026–26/01/2026), when pods were up
3. *node_telemetry_pods_off.csv* contains time-series measurements collected from infrastructure nodes for the second period (31/01/2026–02/02/2026), when pods were down
4. *pod_telemetry_pods_off.csv* contains time-series measurements collected by monitoring the deployed pods for the second period (31/01/2026–02/02/2026), when pods were down

Each row corresponds to a single timestamped observation for a specific node or pod. Telemetry are collected every 30 seconds.

In node telemetry files, columns are structured as presented in Table 1.

**Table 1:** Node_telemetry files’ fields explanation.

Column name	Description	Unit
node_name	Node identifier	vm-node or rpi4
timestamp	Measurement timestamp	DD-MM-YYYY HH:MM:SS
CPU (%)	Node CPU utilization	Percent (%)
MEM (%)	Node memory utilization	Percent (%)
fs (%)	Filesystem utilization of the node	Percent (%)
Energy (watts)	Power consumption of the node	Watts
rx (B/sec)	Incoming network traffic	Bytes per second
tx (B/sec)	Outgoing network traffic	Bytes per second

In pod telemetry files, columns are structured as presented in Table 2.

**Table 2:** Pod_telemetry files’ fields explanation.

Column name	Description	Unit
pod_name	Pod name	weatherforecaster-berlin-edge or weathercollector-berlin or weathercollect-thess or weatherforecaster-berlin-vm or forecaster-tenant-pool
timestamp	Measurement timestamp	DD-MM-YYYY HH:MM:SS
CPU (%)	Pod CPU usage relative to node CPU capacity	Percent (%)
MEM (B)	Pod memory usage out of total node memory	Bytes
Energy (watts)	Energy consumption attributed to the pod	Watts

**Fig. 1:**
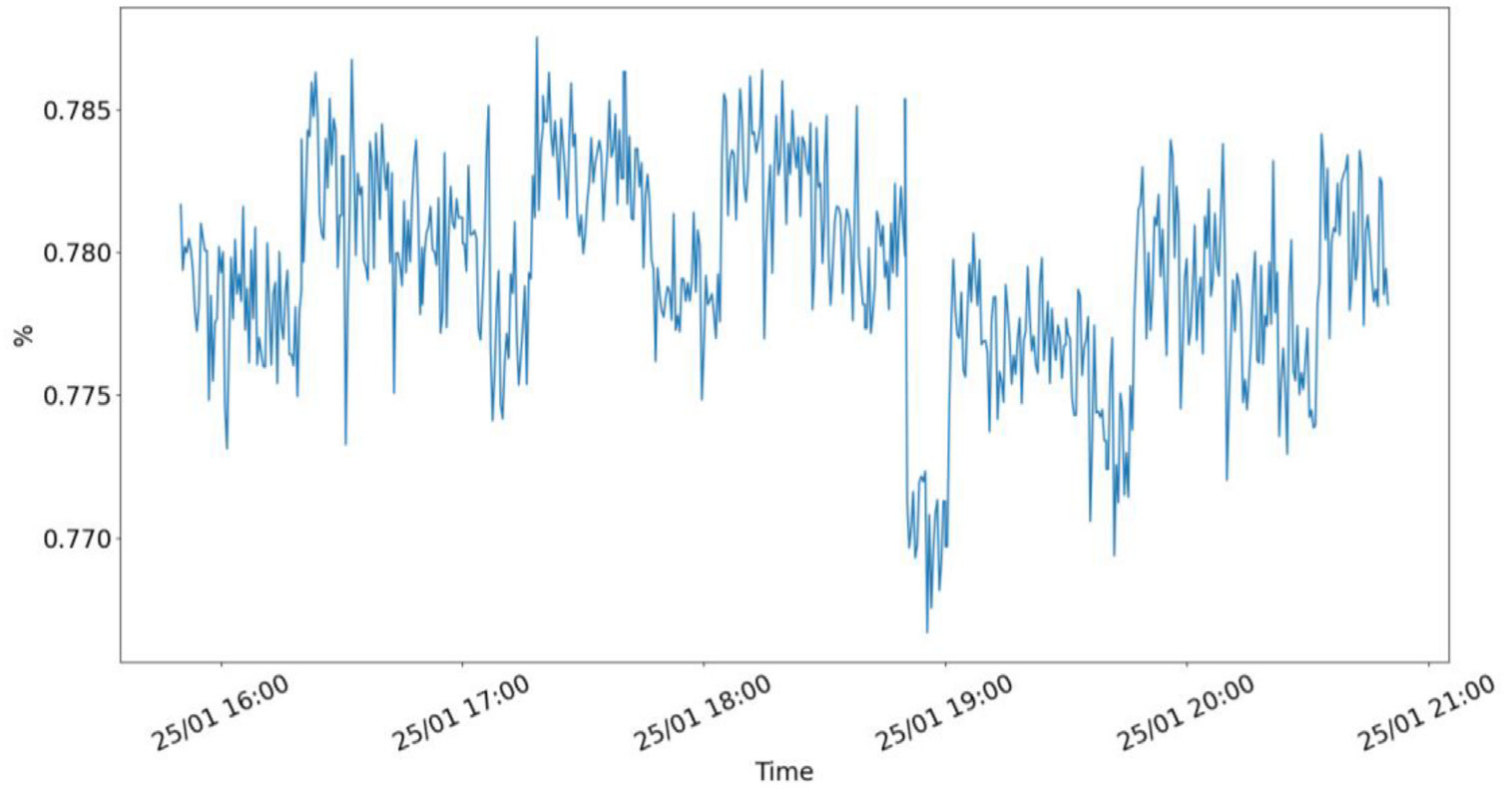
Edge node memory consumption over 4 h when pods are up.

**Fig. 2:**
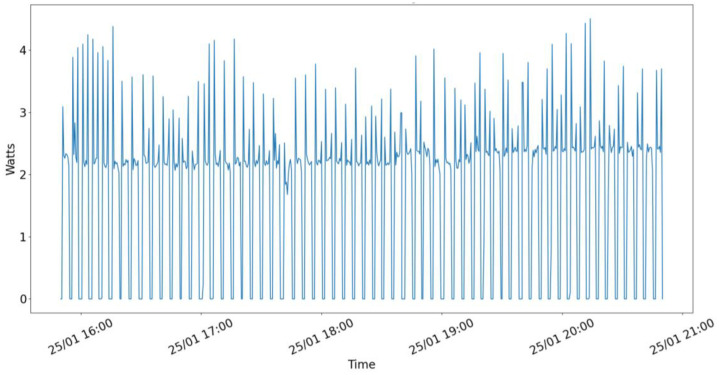
Weather forecaster edge pod energy consumption over 4 h when pods are up.

Two indicative samples of the telemetry are provided in Fig. 1, Fig. 2, presenting the measured memory consumption of the raspberry pi device and the AI-based weather forecaster edge workload from 16:00 to 21:00 at the 25th of January (when the workloads were active).

## 4 Experimental Design, Materials and Methods

Data acquisition was performed in a cluster belonging to the ENACT Cognitive Computing Continuum (CCC), a distributed Kubernetes-based infrastructure designed to emulate realistic edge-cloud continuum scenarios. The cluster operates as a shared environment in which several workloads are executed concurrently across available nodes. For the dataset creation experiment, new workloads were deployed alongside other active services in the cluster; therefore, measurements were obtained under non-isolated, real-world operational conditions rather than in a controlled or dedicated testbed.

**Fig. 3:**
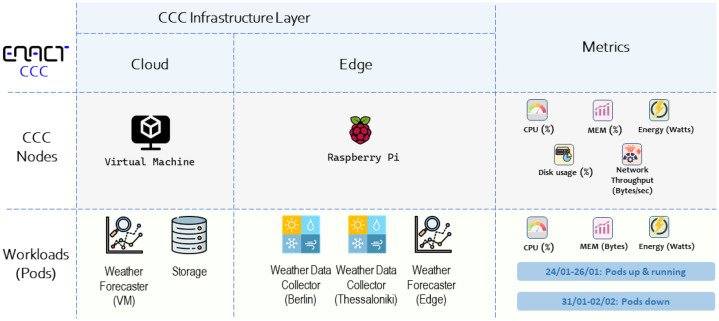
Overview of experimentation setup.

An overview of the experimental setup is depicted in Fig. 3. As depicted, the rows correspond to the categories for which data have been collected, i.e., nodes and application workloads (pods). The first two columns indicate the layer within the ENACT Cognitive Computing Continuum where each node is deployed or each workload is being executed (e.g., a Raspberry Pi device represents the edge node). The rightmost column summarizes the telemetry metrics collected per category, presenting node-level and pod-level measurements, respectively.

More specifically, the setup involved two computing nodes at different tiers of the continuum infrastructure. At the cloud, a Virtual Machine node operates with total computing capacity of 685 millicores (mCPU) and 20,355 MiB of memory, while at the edge, a Raspberry Pi 4 device is equipped with 95 millicores (mCPU) and 1070 MiB of memory. The innate heterogeneity in computational resources between these two nodes enabled the experimentation under diverse computing conditions.

The experiment was based on a distributed weather forecasting business use case. For this purpose, three types of workloads were deployed across the nodes: weather data collectors, AI-based weather forecasting services and a storage service. In particular, two weather data collection pods, each corresponding to a data source from a different city (namely, Thessaloniki and Berlin), were deployed at the edge node. Those weather data collectors are based on the OpenWeather API.^1^ In addition, two instances of an AI-based weather forecasting application were deployed, one at the edge and one at the cloud node. Finally, a MinIO^2^ storage pool was deployed on the cloud node to serve as the persistent storage backend for collected telemetry.

In order to make sure that all pods will be executed normally both at the VM and the Raspberry Pi devices, preventing the edge node from being overwhelmed, appropriate resource limits have been defined, set to 0.25 CPU and 256MiB of memory per pod (for each weather data collector and for the weather forecaster).

Telemetry data were collected using the Telemetry Data Collector and Monitoring Engine (TDCME)^3^ developed within the ENACT project. TDCME integrates several well-established Kubernetes monitoring tools, including Prometheus^4^ for resource utilisation metrics collection, Cilium CNI^5^ for network traffic tracking and Kepler^6^ for measuring energy consumption. Building upon TDCME, telemetry collection was performed continuously in real time, using a sampling interval of 30 seconds. This interval corresponds to the default configuration of TDCME and aligns with the refresh rate of its underlying monitoring components. In particular, network statistics collected via Cilium are updated every 30 seconds, whereas shorter sampling intervals (e.g., 15 secs, as commonly used by Prometheus) resulted in duplicates or negligible differences across the collected metrics (CPU, memory, network, and energy), providing no additional meaningful information while increasing data volume. Therefore, the interval of 30 seconds was selected as the most suitable option to capture telemetry changes in an accurate and efficient manner. Nevertheless, the sampling interval can be configured in TDCME to collect metrics more or less frequently if required.

A snapshot of the measurements was taken every 10 minutes and stored in structured JSON format. Εach JSON file therefore contains time-series telemetry measurements sampled every 30 seconds for the preceding 10 minutes.

Telemetry data included node-level metrics, i.e., CPU, memory and disk utilisation, network throughput (received and transmitted bytes per second), and energy consumption, as well as pod-level metrics, including CPU and memory utilisation, and energy consumption (See Table 1, Table 2 for a detailed overview of recorded metrics). Collected telemetry data are automatically sent to a dedicated bucket within the MinIO storage service. This storage instance is part of ENACT’s Data Space, which provides distributed storage capabilities across the continuum infrastructure.

The dataset comprises telemetry collected during two distinct experimental periods. During the first period, from the 24th of January 2026 to the 26th of January 2026, all pods were deployed and running continuously for three consecutive days. During the second period, from the 31st of January 2026 to the 2nd February 2026, all application pods except for the MinIO storage pod were scaled down to zero replicas. This enabled the observation of baseline system behaviour.

During the first period, the deployed forecasting and weather collection services were executed at predefined intervals. In particular, the weather collector pods retrieve new data every ten minutes, following the update frequency of the source data. This request interval is recommended by the OpenWeather One Call API^1^ in order to receive the most accurate and up-to-date weather data. The AI forecasting services generate predictions every two minutes. This configuration ensured continuous and realistic operational load and allowed for capturing practical resource utilisation and energy consumption telemetry data.

It is important to note that between the two periods there were no changes in the underlying cloud or edge node configurations. The only difference was the presence or absence of the application workloads, which introduced additional load on the nodes during the first period.

The extent of the monitored setup (1 cloud node, 1 edge node and 5 pods) and the experimental duration were a deliberate choice. The scope of the produced dataset was intentionally bounded to provide a public, reusable telemetry release that captures edge–cloud heterogeneity and both loaded and baseline operating regimes, while keeping the volume and complexity manageable for downstream use.

For this reason, telemetry was collected from two nodes representing different tiers of the continuum. Using one node would remove the main comparative dimension of the dataset (no cross-tier heterogeneity), whereas adding more nodes would introduce additional hardware and scheduling variability (extra confounders) and significantly increase operational and data-management overhead without contributing any further to the dataset’s aim for demonstrating the difference between edge and cloud node metrics targeted in this first release. Also, the five deployed pods were selected as the minimum workload for a functionally complete weather forecasting scenario (two collectors, one forecaster per layer and a storage backend). Less pods would weaken the scenario’s lifelikeness, would lead to a loss of telemetry data (e.g., if only one forecaster instance was deployed in one of the computing layers) or would require dropping a key role (e.g., a data source or the data store), while more pods would either replicate existing behavior or add new application types that would shift the scope and require substantially extra configuration/ interpretation effort.

The two experimental periods, i.e., when application pods were up and running and the baseline period, were selected to distinguish application-driven effects from infrastructure behavior. Relying on only the pods-up regime would prevent direct baseline comparison (e.g., overhead estimation, anomaly detection, idle vs loaded characterization). More regimes (e.g., multiple replica counts, partial loads, fault injection) are beyond the intended scope of this release. Each period spans three consecutive days to include at least one full diurnal cycle and repeated variability patterns, without expanding the dataset into long-horizon monitoring where additional duration would mostly add more repetitions of the same regimes. Finally, metrics are provided in 10-minute observation windows, selected as a practical trade-off: shorter windows (e.g., 5 minutes) would markedly increase file/record counts and sensitivity to short-term noise, while longer windows (e.g., 15+ minutes) would oversmooth behavior and risk hiding transient spikes relevant to scheduling and control-loop evaluation.

The collected telemetry JSON files were retrieved from the MinIO storage bucket and processed using custom Python scripts to extract the metrics described in Table 1, Table 2. The outcome is a collection of .csv files to facilitate further analysis and reusability. Each file contains the time-series measurements as recorded every 30 seconds; thus, values correspond to the actual telemetry measurements rather than aggregated statistics (e.g., averages or maximums).

## Limitations

The dataset was collected in a non-isolated cluster, where multiple workloads were running concurrently alongside the monitored pods. As a result, resource metrics recorded at the node level may be influenced by the activity of other services on the same node. However, this ensures that collected telemetry reflects real operational conditions, which was the main goal of this work. Moreover, some metrics, such as energy consumption and network throughput, may be affected by the hardware or overall network contention. In addition, in large-scale IoT environments or edge-cloud deployments, hardware and software heterogeneity are typically higher; in this dataset, telemetry was collected from a single Raspberry Pi edge node and a cloud VM, which although are sufficient for the purposes of this work, limit the representation of such heterogeneity.

## Ethics Statement

The authors have read and follow the ethical requirements for publication in Data in Brief and the current work does not involve human subjects, animal experiments, or any data collected from social media platforms.

## CRediT Author Statement

**Ioanna Angeliki Kapetanidou**: Data curation, Software, Supervision, Writing – original draft. **Thanasis Kotsiopoulos**: Conceptualization, Investigation, Methodology, Validation, Supervision, Writing – original draft**. Giorgos Thanasoulis**: Methodology, Software, Validation, Writing – review & editing. **Paschalis Bizopoulos**: Investigation, Software, Validation, Writing – review & editing. **Athanasios Liatifis**: Software, Writing – review & editing. **Manolis Skoularikis**: Software, Writing – review & editing. **Alexandros Nizamis**: Conceptualization, Funding acquisition, Supervision, Writing – review & editing. **Panagiotis Sarigiannidis**: Funding acquisition, Supervision. **Konstantinos Votis**: Funding acquisition, Supervision.

## Data Availability

(Zenodo).Resource Utilisation and Energy Consumption Telemetry for a Weather Forecasting Scenario in the ENACT Continuum (Original data).

## Footnotes

1 https://openweathermap.org/api/one-call-3?collection=one_call_api_3.0.

2 https://www.min.io/.

3 https://gitlab.eclipse.org/eclipse-research-labs/enact-project/telemetry-data-collector-and-monitoring-engine.

4 https://prometheus.io/.

5 https://cilium.io/.

6 https://sustainable-computing.io/.
